# Transcranial direct current stimulation alleviates the pain severity in people suffering from knee osteoarthritis: a systematic review and meta-analysis

**DOI:** 10.1097/PR9.0000000000001215

**Published:** 2024-12-09

**Authors:** Tian Dai, Meng Liu, Dapeng Bao, Brad Manor, Junhong Zhou

**Affiliations:** a China Institute of Sport and Health Science, Beijing Sport University, Beijing, China; b National Sports Training Center, Beijing, China; c Sports Coaching College, Beijing Sport University, Beijing, China; d School of Physical Education, University of Jinan, Shandong, China; e Medical examination center, Peking University, Third Hospital, Beijing, China; f Hebrew SeniorLife Hinda and Arthur Marcus Institute for Aging Research, Harvard Medical School, Boston, MA, USA

**Keywords:** Transcranial direct current stimulation, Pain, Knee osteoarthritis, Meta-analysis

## Abstract

Considerable research has shown the benefits of transcranial direct current stimulation (tDCS) for the alleviation of pain associated with knee osteoarthritis (KOA). Still, a large variance in study protocols and observations across publications exists. We here thus completed a systematic review and meta-analysis to comprehensively and quantitatively characterize the effects of tDCS on KOA-related pain. A search strategy based on the Population, Intervention, Comparison, Outcome, and Study design (PICOS) principle was used to obtain the publications in 7 databases. Studies exploring the effects of tDCS on KOA-related pain were screened, and eligible studies were included. Ten studies of 518 participants using Visual Analogue Scale or Numeric Rating Scale to assess pain were included in the systematic review, and 9 of them were included in meta-analysis. The quality of these studies was good. Compared to control, tDCS induced significant short-term improvements in KOA-related pain with medium heterogeneity (standardized mean difference [SMD] = −0.91, 95% confidence interval [−1.24, −0.58], *P* < 0.001, I^2^ = 61%). Subgroup analyses showed that both home-based (SMD = −1.32, 95% CI [−1.65, −0.99], *P* < 0.001, *I*^2^ = 0%) and laboratory-based intervention (SMD = −0.66, 95% CI [−0.99, −0.33], *P* < 0.001, *I*^2^ = 40%) with at least 5 sessions per week (SMD = −1.02, 95% CI [−1.41, −0.64], *P* < 0.001, *I*^2^ = 65%) and/or with a total number of at least 10 sessions (SMD = −1.12, 95% CI [−1.51, −0.74], *P* < 0.001, *I*^2^ = 59%) can induce maximum benefits for the alleviation of KOA-related pain. The results here showed that tDCS is of great promise to alleviate KOA-related pain. Still, future studies with more rigorous design are needed to confirm the observations from this work, which can ultimately help the determination of appropriate intervention protocol that can maximize such benefits.

## Abstract 2

Supplemental Digital Content is Available in the Text.

Home- or laboratory-based transcranial direct current stimulation with at least 5 sessions per week and/or with at least 10 sessions can induce significant benefits for knee osteoarthritis–related pain.

## 1. Introduction

Knee osteoarthritis (KOA) is one of the leading causes of pain symptoms with advancing age.^24,29,51^ The KOA-related pain oftentimes induces disabled mobility, increased risk of falls, and mood problems (eg, depression), severely diminishing the functional independence in people suffering from this condition.^39^ It is thus critical to effectively manage the KOA-related pain and alleviate its burden, which will ultimately improve the quality of life in people with KOA.

Numerous strategies have been implemented, such as medications (eg, analgesics, low potency opioids^21,47,49^), injection of chemicals (eg, corticosteroids, hyaluronic acid^7,31^), and total knee arthroplasty surgery.^22^ However, these strategies oftentimes are expensive and with side effects that cannot be ignored (eg, the incidence of nausea, drowsiness, and constipation after the therapy of analgesics or opioids).^20,48,52^ Recent evidence has linked the activation of supraspinal regions/networks to pain.^18,25,40,42^ For example, Parker et al.,^42^ report in a systematic review and meta-analysis that compared to the control, the excitability of the primary motor cortex (M1) in people suffering from chronic pain was disinhibited. Therefore, strategies with the goal to modulate the excitability of those supraspinal regions/networks may help alleviate the pain in people suffering from KOA.

One such strategy is transcranial direct current stimulation (tDCS).^37^ Transcranial direct current stimulation is a noninvasive brain stimulation technique that can modulate the cortical excitability of the brain by delivering low-intensity direct currents via the electrodes that are placed over the scalp. Studies have shown the promise of using tDCS targeting cortical sensorimotor regions (eg, M1) for the alleviation of KOA-related pain. However, inconsistent observations have been reported in those studies. For example, Ahn et al., observed significant reductions in the severity of KOA-related pain as induced by tDCS targeting M1 compared to sham^1^; but in another study, Azizi et al., observed no such significant benefits of tDCS targeting M1 for KOA-related pain. These inconsistent observations may be due to the variance in the design of study protocols (eg, participant characteristics, tDCS protocol) across those studies. More recently, several meta-analyses^10,13,57^ have characterized the efficacy of using tDCS on the alleviation of KOA-related pain. Yang et al.,^57^ for example, showed that compared to sham, tDCS can induce significant decrease of pain severity in people with KOA; in another meta-analysis by Dissanayaka et al.,^13^ it showed that tDCS can be considered as standalone and an adjunct treatment for pain alleviation in people with KOA. However, the contributions of many important characteristics, such as the participant age, stimulation environment (ie, home-based or laboratory/clinical-based), to such benefits of tDCS for KOA-related pain have not been explicitly characterized.

Therefore, we here performed a systematic review and meta-analysis consisting of several exploratory subgroup analyses based upon the most up-to-date available peer-reviewed publications to quantitatively examine the efficacy of tDCS on KOA-related pain. The knowledge obtained from this work will ultimately help inform the appropriate design of future management programs and rehabilitative strategies using tDCS to alleviate KOA-related pain.

## 2. Methods

### 2.1. Design

This systematic review and meta-analysis was conducted in accordance with the Preferred Reporting Items for Systematic Reviews and Meta-Analyses (PRISMA)^41^ and registered with PROSPERO (Registration ID CRD42022347999).

### 2.2. Literature search

Seven electronic databases (PubMed, EMBASE, MEDLINE, CINAHL Complete,^54^ SPORTDiscus, Web of Science, and Cochrane Library) were used to search articles. Databases were searched from the initial date of each dataset to March 1, 2024. Searches were limited to publications in English only. The PICOS (Population, Intervention, Comparison, Outcome, and Study design) framework was used to develop and refine the search strategy. The following search terms were used to identify relevant literature in the databases: (“transcranial direct current stimulation” OR “tDCS”) AND (“pain” OR “pain symptom” OR “painful symptom”) AND (“knee osteoarthritis” OR “knee arthritis” OR “gonitis” OR “gonarthritis”). A manual search of the bibliographic references for extracted articles and existing reviews was conducted to identify studies that were not captured in the database searches.

### 2.3. Inclusion and exclusion criteria

The inclusion criteria were (1) the intervention type was tDCS alone or tDCS in combination with other treatments (eg, mindfulness-based meditation [MBM], muscle electrical stimulation [EIMS], or exercise) against a control group using sham stimulation; (2) the outcome measures were related to pain symptom (eg, pain intensity measured via Visual Analogue Scale [VAS] or Numeric Rating Scale [NRS]); and (3) the design of the study was parallel or cross-over randomized controlled trial.

The exclusion conditions included (1) repeated publication; (2) system review, case report, protocol papers, conference abstract and papers, letters to editor; (3) not written in English; (4) unable to obtain outcome data; (5) tDCS combined with other types of brain stimulation (eg, transcranial magnetic stimulation); and (6) copresence of hip and knee osteoarthritis.

### 2.4. Data extraction

Two authors (T.D. and M.L.) independently performed data extraction, and when the disagreement on decision happened, it was discussed with other authors (D.B. and J.Z.) until a consensus was achieved. We extracted the relevant information of each study, including authors, publication year, characteristics of participants (eg, age, gender and sample size) and interventions (type, frequency, number of sessions, time length of each session, intervention duration), outcome measures, the design of tDCS (ie, placement, polarity and size of the electrodes, current intensity, length of stimulation, stimulation target, and weekly frequency), and information of participant tolerance and side/adverse effects.

For each included study, the mean and standard deviation of changes from preintervention to postintervention were extracted. Any outcome measures on which the 2 authors disagreed was discussed with a third author (D.B. or J.Z.) until a consensus was achieved. If these values were not available, they were calculated using the following formulas, where the correlation coefficient (Corr) was assumed at 0.5, which was a conservative estimate between 0 and 1 following the principles of the Cochrane Handbook for Systematic Reviews of Interventions,^19,56^ ${\text{SD}}_{change}=\sqrt{{\text{SD}}_{\text{pre}}^{2}+{\text{SD}}_{\text{post}}^{2}-2\times \text{Corr}\times {\text{SD}}_{\text{pre}}\times {\text{SD}}_{\text{post}}}$. If any relevant data were missing, we contacted the authors via email.

### 2.5. Quality assessment

The quality of methodologies in included studies was quantified by the Physiotherapy Evidence Database (PEDro) scale. The PEDro scale included 11 items, and each study was assessed as either “1” (criterion is satisfied) or “0” (criterion not satisfied) for each of those items. According to the PEDro guidelines, the maximum total score was 10 (item 1 is not used to compute the total score), and the higher the score, the greater the study quality. The score of excellent quality was 9 to 10, good quality was 6 to 8, normal quality was 4 to 5, and poor quality was lower than 4. Methodological quality was independently assessed by 2 authors (T.D. and M.L.). Any score on which the 2 authors disagreed was discussed with a third author (J.Z. or D.B.) until a consensus was achieved. The quality of the evidence was also assessed independently by 2 authors (T.D. and M.L.) based on the GRADE criteria.^3,4^ Any score on which the 2 authors disagreed was discussed with a third author (J.Z. and D.B.) until a consensus was achieved.

### 2.6. Statistical analysis

Considering that both the NRS and VAS were widely used and validated scales to assess pain severity, we selected both as primary endpoints for this study. We then used the Bonferroni correction to account for the multiple comparisons, and the significance level for each test was adjusted to *P* = 0.025 (0.05/2). Due to the outcome being reported using different measurement units, continuous data were analyzed by combining the standardized mean difference (SMD) of each outcome across studies. For any missing values, we used the mean imputation method, that is, using the mean of the dataset to replace the missing values. To determine the effect size (ES) of the intervention, the SMD of the outcomes was calculated, with a 95% confidence interval (CI). The ES was obtained by first calculating the difference from the mean pre–post change in the intervention group to the mean pre–post change in the control group, and dividing this difference by the pooled pretest SD. According to Cohen standards, effect sizes were classified as trivial (<0.2), small (0.2–0.5), moderate (0.5–0.8), or large (>0.8). Meta-analysis was performed in Review Manager (RevMan version 5.4, Cochrane Collaboration, Oxford, United Kingdom) using the inverse variance method for included studies that compared the effects of tDCS and control conditions on each included outcome. Heterogeneity was assessed using the chi-squared (χ^2^) and *I*^2^ values. The level of heterogeneity was interpreted according to guidelines from the Cochrane Collaboration, that is, *I*^2^ values of 25%, 50%, and 75% corresponded to low, moderate, and high heterogeneity, respectively. A random-effect model was used to estimate the pooled effect in anticipation of heterogeneity across the studies due to differences in participants and intervention characteristics. In addition, publication bias was assessed by generating funnel plots and conducting Egger test. If a significant asymmetry was detected, we used Trim and Fill method for sensitivity analysis of the results.^15^

Exploratory subgroup analysis was performed to examine the influence of several factors (eg, number of stimulation sessions) on the outcomes.

## 3. Results

### 3.1. Study selection

**Figure 1.**
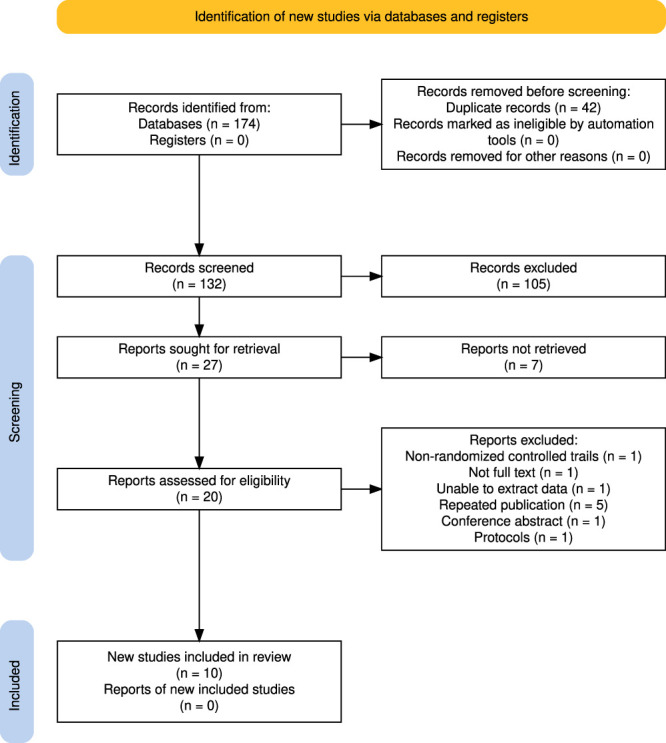
Selection process.

The study identification and selection process were summarized in Figure 1. The initial search identified 174 potentially relevant articles. A total of 42 duplicate publications were excluded, leaving 132 articles. Then, 105 irrelevant articles were excluded by title and abstract, and 27 publications with full text remained. Next, these 27 full-text articles were evaluated for eligibility, and 7 of them were excluded. These 20 articles were further evaluated by reviewing the whole article, and 10 of them were excluded. Therefore, a total of 10 publications were included in this work (Table 1).

**Table 1:** Characteristics of included studies.

Author/year; country	Sample size	Mean age (y)	Gender	Raw NRS/VAS scores	Intervention	Follow up time points	tDCS vs control group outcome measures	Main findings	tDCS device specifics
Martorella et al.^30^ (2022); USA	tDCS (n = 60)	65.32 (8.41)	82F, 38M	NRS: 55.05 (21.96)	Current intensity: 2 mA electrode size: 5 cm × 7 cm Anode: primary motor cortex (M1) Cathode: supraorbital region (SO) 20 minutes × 5 times/week × 3 wk tDCS: gradually ramped up (to 2 mA) and down (to 0 mA) during 30 seconds at the beginning and end of the stimulation	1, 2, and 3 mo from baseline	End of treatment NRS: ↑, *P* < 0.0001* WOMAC: ↓, *P* = 0.10 end of follow-up (3 mo from baseline) NRS: ↑, *P* < 0.01* WOMAC:↓, *P* = 0.56	Home-based tDCS with real-time remote supervision was associated with significant improvement in clinical pain intensity up to 3 mo after a 3-week treatment	1 × 1 tDCS mini-CT Stimulator device (Soterix Medical Inc, NY), saline-soaked surface sponge electrodes
	Sham-stimulation (n = 60)	66.60 (8.43)		NRS: 50.63 (21.77)	Sham-stimulation: in the same positions and stimulator only delivered 2 mA current for 30 s				
Tavares et al.^50^ (2021);Brazil	tDCS (n = 51)	74.78 (7.44)	88F, 16M	VAS: 6.03 (1.52)	Current intensity: 2 mA electrode size: 35 cm^2^ Anode: M1; cathode: SO 20 minutes × 5 times/week × 3 weeks tDCS: gradually ramped up (to 2 mA) and down (to 0 mA) during 30 seconds at the beginning and end of the stimulation	15 d, 1 and 2 mo after the treatment end	End of treatment Pain score: BPI: ↑, *P* < 0.001* VAS pain: ↑, *P* < 0.005* SF-12: PCS:↓, *P* = 0.52; MCS:↓, *P* = 0.77 WOMAC total score: ↓,*P* = 0.81 Lequesne Index total score: ↓,*P* = 0.95 BPI function interference: ↓,*P* = 0.86 OLS, seconds: ↓,*P* = 0.34 VAS self-rated health: ↓,*P* = 0.33 Von-frey NRS: Knee:↓, *P* = 0.83; Hand: ↓,*P* = 0.19 PPT: Knee: ↓,*P* = 0.14; Hand: ↓,*P* = 0.66 CPM PPT: Knee: ↑,*P* = 0.01*; Hand: ↓,*P* = 0.57 CPM P: Knee:↓, *P* = 0.86; Hand:↑, *P* = 0.01* end of follow-up (2 months after the treatment end) Pain score: BPI ↓,*P* = 0.08 VAS pain: ↓,*P* = 0.70 SF-12: PCS: ↓,*P* = 0.49; MCS:↓, *P* = 0.16 WOMAC total score: ↓,*P* = 0.56 Lequesne Index total score: ↓, *P* = 0.133 BPI function interference: ↓,*P* = 0.38 OLS, seconds:↓, *P* = 0.31 VAS self-rated health: ↓,*P* = 0.22 Von-frey NRS: Knee: ↓,*P* = 0.28; Hand: ↓,*P* = 0.22 PPT: Knee: ↓,*P* = 0.70; Hand: ↓, *P* = 0.72 CPM PPT: Knee: ↓, *P* = 0.28; Hand: ↓, *P* = 0.27 CPM P: Knee: ↓, *P* = 0.22; Hand: ↓, *P* = 0.27	M1-SO tDCS were effective and safe to reduce pain and to improve the DPIS function in elderly patients with KOA chronic pain, these effects were not sustained over the 2-mo follow-up period	tDCS equipment (EASYpad™ Soterix Medical Inc), 2 surface sponge electrodes soaked with physiologic saline
	Sham-stimulation (n = 53)	73.13 (8.51)		VAS: 6.11 (1.47)	Sham-stimulation: same electrodes location as active tDCS and only included the 30 seconds of each ramp-up/down periods, mimicking stimulation sensations of active tDCS				
Rahimi et al.^45^ (2021);Iran	tDCS on M1 +PT (n = 20)	58.8 (3.3)	36F, 4M	VAS: 9.35 (0.75)	Current intensity: 1 mA Electrode size: 4 × 4 cm Anode: M1 or S1 or DLPFC; cathode: SO 20 minutes × 5 times/week × 2 weeks tDCS: gradually ramped up (to 1 mA) and down (to 0 mA) during 30 seconds at the beginning and end of the stimulation PT: 10 sessions (5 sessions per week) and included transcutaneous electrical nerve stimulation (TENS), ultrasound, heat (infrared), patellofemoral and tibiofemoral mobilization (grade 1 and 2) and exercise therapy	1 mo after the treatment end	M1 tDCS + PT vs Sham-stimulation + PT end of treatment VAS: ↑, *P* < 0.001* KOOS: ↑, *P* < 0.001* ROM: ↓, *P* = 0.868 Quadriceps femoris strength (Nm): ↓, *P* = 0.902 Stepping (n of steps): ↑, *P* = 0.028* Chair stand (n of standings):↑, *P* = 0.002* 10 m-walking (time in s): ↓, *P* = 0.107 end of follow-up (1 mo after the treatment end) VAS: ↑, *P* < 0.001* KOOS: ↑, *P* < 0.001* ROM: ↓, *P* = 0.859 Quadriceps femoris strength (Nm): ↑, *P* = 0.012* Stepping (n of steps): ↑, *P* < 0.001* Chair stand (n of standings): ↑, *P* < 0.001* 10m-walking (time in s): ↑, *P* < 0.001*	The results indicated a positive effect of adding tDCS to PT, on patient pain and physical performance	tDCS equipment (OASIS Pro, Mind Alive Inc, Canada), saline-soaked surface electrodes
	tDCS on S1 +PT (n = 20)			VAS: 9.30 (0.73)					
	tDCS on DLPFC + PT (n = 20)			VAS: 9.55 (0.60)					
	Sham-stimulation + PT (n = 20)			VAS: 9.35 (0.75)	Sham-stimulation: Current intensity rose from 0 mA to 1 mA for 30 seconds, and then the current was turned off PT: same intervention				
Azizi et al.^5^ (2021); Iran	tDCS (n = 27)	61.3 (13.5)	39 F, 15 M	VAS: 6.4 (2.3)	Current intensity: 2 mA Electrode size: 5 × 7 cm Anode: M1; cathode: SO 20 minutes × 5 times/week × 1 week tDCS: gradually ramped up (to 2 mA) and down (to 0 mA) during 10 seconds at the beginning and end of the stimulation	3 mo after the treatment end	End of follow-up (3 mo after the treatment end) VAS: ↓, *P* = 0.226 KOOS: Symptoms: ↓, *P* = 0.680; Pain: ↑, *P* = 0.007*; Daily living: ↑, *P* = 0.009*; Sports: ↑, *P* = 0.003*; Quality of life: ↑, *P* < 0.001*; Total: ↑, *P* = 0.005* No other raw data are presented in the original text	tDCS did not affect the symptoms of KO. Only a small favorable change in VAS was recognized for both sham and active groups	Endomed device (ENRAF Company, The Netherland, Amsterdam), sponge electrodes soaked in a 1% saline solution
	Sham-stimulation (n = 27)	56.4 (11.7)		VAS: 5.8 (1.5)	Sham-stimulation: in the same positions and 10 seconds ramp-up of the current from zero to 2 mA, 30 seconds stimulation, 10 seconds ramp down to zero, and 20 minutes no current				
Sajadi et al.^46^ (2020); Iran	tDCS + exercise (n = 20)	59.30 (6.13)	33 F, 7 M	VAS: 68.50 (19.54)	Current intensity: 2 mA Electrode size: 35 cm^2^ Anode: M1; cathode: SO 20 minutes × 3 times/week × 2 weeks tDCS: a constant current with an intensity of 2 mA was applied for 20 minutes per session Exercise: Closed chain strengthening exercise of the quadriceps muscle (eg, wall squat, forward step-up, lunges), and patients were told to do the mentioned exercises twice a day during the treatment period	1 wk, 1 mo and 3 mo after the treatment end	VAS:↓, *P* = 0.263 WOMAC: total:↓, *P* = 0.051; stiffness: ↓, *P* = 0.198; pain: ↓, *P* = 0.075; Function: ↓, *P* = 0.146 The *P*-value for group and time interaction	Both TENS and tDCS combined with exercise improved patients' knee pain and functional condition toward 1 mo after the end of treatment and their improving effect was preserved for another 2 mo. Furthermore, the early improvement in patients' pain and function was significant only in patients who received tDCS.	Battery-driven stimulator (Activadose II), rubber electrodes covered with sponges soaked in 0.9% saline
	TENS + exercise (n = 20)	56.85 (5.81)		VAS: 71.00 (15.18)	TENS: 25 minutes × 3 times/week×2 weeks positioned 4 rubber electrodes with a surface of 35cm2 in a square pattern around the kneecap and electrical current was applied to the most painful part of the affected knee (approximately 5 cm apart, centered over the pain point, frequency of 100 Hz, pulse width of 100 seconds)				
Pollonini et al.^44^ (2020); USA	tDCS + MBM (n = 15)	60 (7.7)	N/A	NRS	Current intensity: 2 mA Electrode size: 5 × 7 cm Anode: M1; cathode: SO 20 minutes × 5 times/week × 2 weeks	N/A	NRS: ↑, *P* < 0.001* WOMAC: ↓, *P* = 0.11	The combination of tDCS and meditation had a beneficial effect on the perception of OA-related pain measured with NRS scale	1 × 1 tDCS mini-CT Stimulator (Soterix Medical Inc., NY), saline-saturated sponge electrodes
	Sham-stimulation + MBM (n = 15)				The same location as tDCS and stimulator delivered no electrical current except a ramp-up/down period of 30 s at the beginning and end of trials				
da Graca-Tarragó et al.^12^ (2019); Brazil	tDCS + sham-EIMS (n = 15)	64.14 (9.82)	60 F	VAS: 6.57 (0.51)	Current intensity: 2 mA Electrode size: 5 × 7 cm Anode: M1; cathode: SO 30 minutes × 5 times/week × 1 week tDCS: with an intensity of 2 mA, ramp-up and ramp-down duration of 30 s	N/A	VAS: ↑, *P* = 0.03*; PPT: ↑, *P* = 0.02*; WOMAC: ↑, *P* = 0.03*; BDNF: ↓, *P* = 0.58	Novel approach to applying a combined active interventions tDCS and EIMS over the M1 improved pain scores and the DPMS function. Also, it improved the disability and reduced analgesic use	Not mentioned, rubber electrodes were attached to a sponge soaked in 0.9% saline
	Sham-stimulation + sham-EIMS (n = 15)	63.87 (7.07)		VAS: 6.73 (0.46)	Sham-stimulation: performed the same way as the active stimulation but the tDCS device was prepared to turn off after 30 s of ramp-up				
	tDCS + EIMS (n = 15)	66.00 (9.08)		VAS: 6.31 (1.14)					
	Sham-stimulation + EIMS (n = 15)	64.40 (6.02)		VAS: 6.07 (1.39)					
Ahn et al.^2^ (2019); USA	tDCS + MBM (n = 15)	59.47 (6.38)	18 F, 12 M	NRS: 55.33 (21.00)	Current intensity: 2 mA Electrode size: 5 × 7 cm Anode: M1; cathode: SO 20 minutes × 5 times/week × 2 weeks tDCS: no details about stimulation are given	N/A	NRS: ↑, *P* < 0.0001* WOMAC: ↑, *P* = 0.02* PPT lateral knee:↑, *P* < 0.001* PPT medial knee:↑, *P* < 0.0001* CPM: ↑, *P* < 0.0001* Cold pain: ↑, *P* < 0.01*	All the outcome measures (clinical pain, OA-related clinical symptoms, and quantitative sensory testing measures) were significantly better in the intervention group (home-based tDCS paired with MBM) than in the sham group	1 × 1 tDCS mini-CT Stimulator (Soterix Medical Inc., NY), saline-soaked surface sponge electrodes
	Sham-stimulation + MBM (n = 15)	59.47 (7.63)		NRS: 40.67 (13.35)	Sham-stimulation: The same location as tDCS and stimulator delivered no electrical current except a ramp-up/down period of 30 s at the beginning and end of trials				
Chang et al.^9^ (2017); Australia	tDCS + exercise (n = 15)	59.8 (9.1)	20 F, 10 M	VAS: 59.8 ± 15.2 (pain on walking)	Current intensity: 1 mA Electrode size: 35 cm^2^ Anode: M1; cathode: SO 20 minutes × 2 times/week × 8 weeks tDCS: Current intensity was ramped up (0 mA to 1 mA) and down (1 mA to 0 mA) over 10 s at the beginning and end of the stimulation period	N/A	VAS: ↑, *P* < 0.001* Physical function: ↑, *P* = 0.01* PPT:↑, *P* < 0.05*	The preliminary evidence indicates that adding tDCS to exercise may be a promising approach for improving pain, physical function, and pain mechanisms in knee OA.	Direct current stimulator (DC-STIMULATOR, neuroConn, Ilmenau, Germany), surface sponge electrodes
	Sham-stimulation + exercise (n = 15)	64.1 (11.1)		VAS: 56.4 ± 19.7 (Pain on walking)	Sham-stimulation: The same position as tDCS and turned on for 15 s, then off, to provide the initial itching sensation (participants were informed that they may or may not perceive any sensation during stimulation)				
Ahn et al.^1^ (2017); USA	tDCS (n = 20)	60.6 (9.8)	21 F, 19 M	NRS: 27.30 (15.00)	Current intensity: 2 mA Electrode size: 5 × 7 cm Anode: M1; cathode: SO 20 minutes × 5 times/week × 1 week tDCS: with a constant current and electrical current was ramped up and ramped down over 10 seconds at the beginning and end of the stimulation period, respectively	1, 2, and 3 wk after the treatment end	End of treatment NRS: ↑, *P* = 0.01* WOMAC change Pain: ↓, *P* = 0.45;Stiffness: ↓, *P* = 0.33; Functional impairment: ↓, *P* = 0.39 SF-MPQ-2 change Continuous pain: ↓, *P* = 0.07; Intermittent pain: ↓, *P* = 0.09; Neuropathic pain: ↓, *P* = 0.41 Affective description:↓, *P* = 0.06 SPPB change: ↓, *P* = 0.41 6WMT change: ↓, *P* = 0.10 end of follow-up (3 wk after the treatment) NRS: ↑, *P* = 0.02*	Significant reductions in NRS-rated knee pain in the active tDCS group compared to the sham tDCS group	Not mentioned, a pair of thick (0.3 cm) surface sponge electrodes saturated with 10 cc of saline
	Sham-stimulation (n = 20)	59.3 (8.6)		NRS: 19.00 (7.70)	Sham-stimulation: in the same position and stimulator delivered 2 mA of current for only 30 seconds, with the same ramp up and down period of 10 seconds				

↑: alleviation of pain and improvement in performance, ↓: more severe pain or poorer performance.

* Statistical difference was reported.

6MWT, 6-Minute Walk Test; BDnF, brain-derived neurotrophic factor; BPI, brief pain inventory; CPM, conditioned pain modulation; HPTh, heat pain threshold; HPTo, heat pain tolerance; HSS, hospital for special surgery; KOOS, knee injury and osteoarthritis outcome score; MCS, mental component summary; NRS, numeric rating scale; OLS, one leg stance test; PCS, physical component summary; PPT, pain pressure threshold; SF-12, 12-Item Short-Form Health Survey scale; SF-MPQ, Short-Form McGill Pain Questionnaire; SPPB, short physical performance battery; VAS, visual analogue scale; WOMAC, Western Ontario and McMaster Universities Osteoarthritis Index.

### 3.2. Quality assessment

It was demonstrated that all 10 studies were scored between 7 and 10, suggesting the quality was good (Table 2). The experimental design of 5 studies did not adapt allocation concealment or provide a detailed description of it. For example, the phrase “the allocation concealment was ensured”^2^ lacks adequate detail regarding allocation concealment. Only when it is stated that “the allocation sequence was concealed in consecutively numbered, sealed opaque envelopes”^50^ can it be deemed successful allocation concealment. Eight studies were double blinded^1,2,9,12,30,44,45,50^ that both participants and assessors were blinded to the stimulation type. One study comparing the effects between tDCS and transcutaneous electrical nerve stimulation (TENS) targeting the most painful part of the affected knee stated that it used double-blinded protocol. However, no specific information on blinding protocol was provided in this scenario of using 2 different types of stimulation device with different stimulation target.^46^ Another study was single blinded^5^ (ie, only participants were blinded to the stimulation type). The quality of evidence for the primary outcome (pain severity) based on the GRADE criteria was low (Supplementary Table 1, http://links.lww.com/PR9/A266).

**Table 2:** Quality assessment of included studies.

Study	Eligibility criteria	Random allocation	Concealed allocation	Similarity baseline	Subject blinding	Therapist blinding	Assessor blinding	>85% retention	Intention-to-treat	Between-group comparison	Point and variability measures	Total score
Martorella et al.^30^ (2022)	1	1	0	1	1	0	1	1	1	1	1	8
Tavares et al.^50^ (2021)	1	1	1	1	1	0	1	1	1	1	1	9
Rahimi et al.^45^ (2021)	1	1	0	1	1	0	1	1	1	1	1	8
Azizi et al.^5^ (2021)	1	1	0	1	1	0	0	1	1	1	1	7
Sajadi et al.^46^ (2020)	1	1	1	1	0	0	0	1	1	1	1	7
Pollonini et al.^44^ (2020)	1	0	0	1	1	0	1	1	1	1	1	7
da Graca-Tarragó et al.^12^ (2019)	1	1	1	1	1	0	1	1	1	1	1	9
Ahn et al.^2^ (2019)	1	1	0	1	1	0	1	1	1	1	1	8
Chang et al.^9^ (2017)	1	1	1	1	1	1	1	1	1	1	1	10
Ahn et al.^1^ (2017)	1	1	1	1	1	0	1	1	1	1	1	9

### 3.3. Characteristics of included studies

#### 3.3.1. Participant characteristics

A total of 518 participants were included. Demographics and clinical characteristics of these participants were presented in Tables 1 and 3. The sample size in the included studies ranged from 15 to 60. The mean age was between 56.4 and 74.78 years. All participants were reported to not have previous experience with tDCS or other intervention. One study^12^ recruited only women (n = 30), one study^44^ did not report the number of men and women, and other 8 studies^1,2,5,9,30,45,46,50^ investigated both men (n = 121) and women (n = 337). In 9 studies,^1,2,5,9,30,44–46,50^ participants had symptomatic KOA as determined using American College of Rheumatology (ACR) criteria, and the other one study^12^ did not report relevant information. Seven studies^1,5,12,30,45,46,50^ used Kellgren–Lawrence radiologic criteria (K–L) to assess the severity of KOA in participants, that is, one study^5^ included participants with mild KOA (ie, grades 1–2 based on the K–L), 2 studies^45,46^ included those with moderate KOA (ie, grades 2–3), one study^12^ included participants with serious KOA (ie, grades 3–4), and 3 studies^1,30,50^ included participants with different severity of KOA (ie, grades 1–4). The other 3 studies^2,9,44^ did not report relevant information. Three studies^5,45,46^ recruited participants from hospital or rehabilitation center, one^12^ recruited by specialist referrals and posters, and the other 6^1,2,9,30,44,50^ recruited from the local community.

**Table 3:** Inclusion and exclusion criteria of participant.

Study	Intervention	Age	Pain severity	History of pain (at least)	Prescription being used	ACR criteria	K/L criteria	Exclusion
Martorella et al.^30^ (2022)	a-tDCS/s-tDCS	50–85	30 on 0–100 NRS	3 mo	Had no plan to change medication regimens	3 out of the 6 following features	Grades 1–4	Prosthetic knee replacement or nonarthroscopic surgery to the affected knee
Tavares et al.^50^ (2021)	a-tDCS/s-tDCS	≥60	4 on a 0–10 NRS	6 mo	Do not change the current prescription	N/A	Grades 1–4	N/A
Rahimi et al.^45^ (2021)	a-tDCS/s-tDCS	50–65	4 on a 0–10 VAS	N/A	N/A	3 out of the 6 following features	Degree 2 or 3	Recently received treatment or rehabilitation for KOA
Azizi et al.^5^ (2021)	a-tDCS/s-tDCS	30–70	N/A	3 mo	N/A	N/A	Degree 1 or 2	History of surgery on the knee joint or major trauma to the knee region
Sajadi et al.^46^ (2020)	a-tDCS + exercises/a-TENS + exercises	>50 y	40 on a 0–100 VAS	6 mo	N/A	N/A	Grades II and II	History of knee surgery
Pollonini et al.^44^ (2020)	a-tDCS + MBM/s-tDCS + s-MBM	50–85	30 on 0–100 NRS	3 mo	N/A	3 out of the 6 following features	N/A	N/A
da Graca-Tarragó et al.^12^ (2019)	a-tDCS + s-EIMS/s-tDCS + s-EIMS	50–75	N/A	6 mo	N/A	N/A	Grading of 3–4 K/L	History of knee surgery
Ahn et al.^2^ (2019)	a-tDCS + MBM/s-tDCS + s-MBM	50–85	30 on 0–100 NRS	3 mo	N/A	3 out of the 6 following features	N/A	N/A
W. J. Chang et al. (2017)	a-tDCS + exercise/s-tDCS + exercise	50–75	40 on a 0–100 VAS	Pain on walking in the past week	Continue their usual medications for the duration of the trial	3 out of the 6 following features	N/A	N/A
Ahn et al.^1^ (2017)	a-tDCS/s-tDCS	50–70	N/A	N/A	N/A	N/A	Grades 0–4	Prosthetic knee replacement or nonarthroscopic surgery to the affected knee

ACR criteria, American College of Rheumatology criteria; EIMS, intramuscular electrical stimulation; K/L criteria, Kellgren–Lawrence radiologic; MBM, mindfulness-based meditation; NRS, numeric rating scale; tDCS, transcranial direct current stimulation; VAS, visual analogue scale.

The severity of pain for the inclusion of participants was assessed using NRS in 4 studies^1,2,30,44^ and VAS in 6 studies.^5,9,12,45,46,50^ Specifically, 3 studies^2,30,44^ included participants with a score of at least 30 of NRS with the range from 0 to 100, one study^1^ recruited participants who had at least 10 of NRS (0–100), two studies^9,46^ recruited participants who had at least 40 of VAS (0–100), and 4 studies^5,12,45,50^ recruited participants who had at least 4 of VAS (0–10). The history of KOA-related pain ranged from 3 months (number of studies, n = 4)^2,5,30,44^ to 6 months (n = 3)^12,46,50^ as reported in 7 studies, and uniquely, the participants in one study^9^ experienced KOA-related pain during walking within the past 7 days. In one study,^46^ participants were asked to mark the maximum pain severity they had experienced in the last 2 days using VAS. Such information was not reported in the other 2 studies.^1,45^ Seven studies^1,2,5,9,12,45,50^ included patients with unilateral or bilateral symptoms and the other 3 studies^30,44,46^ did not report relevant information.

#### 3.3.2. Transcranial direct current stimulation characteristics

##### 3.3.2.1. Stimulation target

Table 4 showed the characteristics of tDCS intervention. The M1–SO montage, that is, the anode electrode placed over the primary motor cortex (M1) and the cathode electrode placed over the supraorbital region (SO), was used in all the studies. Specifically, the anode electrode was placed over C3 or C4 of the 10 to 20 system of electroencephalography, to target the hemisphere contralateral to the side with KOA-related pain, or to the side with more severe pain if both sides were with KOA-related pain. Uniquely, one study^5^ reported that if the pain severity was the same between both sides, anodal was placed contralateral to the side of dominant leg in each participant.

**Table 4:** Characteristics of transcranial direct current stimulation.

Study	Position of anode	Position of cathode	Electrode size (cm^2^)	Intensity (mA)	Duration (min)	Weekly frequency	Duration (wk)	No. of sessions	Home-based or laboratory/clinical-based intervention	tDCS-only or tDCS combined with other interventions	Follow-up
Martorella et al.^30^ (2022)	M1	SO	35	2	20	5	3	15	Home-based	tDCS-only	3 mo
Tavares et al.^50^ (2021)	M1	SO	35	2	20	5	3	15	Laboratory/clinical-based	tDCS-only	15 d 1 mo 2 mo
Rahimi et al.^45^ (2021)	M1	SO	16	1	20	5	2	10	Laboratory/clinical-based	tDCS combined with other intervention	1 mo
Azizi et al.^5^ (2021)	M1	SO	16	2	20	5	1	5	Laboratory/clinical-based	tDCS-only	3 mo
Sajadi et al.^46^ (2020)	M1	SO	35	2	20	3	2	6	Laboratory/clinical-based	tDCS combined with other intervention	1 mo 3 mo
Pollonini et al.^44^ (2020)	M1	SO	35	2	20	5	2	10	Home-based	tDCS combined with other intervention	N/A
da Graca-Tarragó et al.^12^ (2019)	M1	SO	35	2	30	5	1	5	Laboratory/clinical-based	tDCS combined with other intervention	N/A
Ahn et al.^2^ (2019)	M1	SO	35	2	20	5	2	10	Home-based	tDCS combined with other intervention	N/A
Chang et al.^9^ (2017)	M1	SO	35	1	20	2	8	16	Laboratory/clinical-based	tDCS combined with other intervention	N/A
Ahn et al.^1^ (2017)	M1	SO	35	2	20	5	1	5	Laboratory/clinical-based	tDCS-only	1 wk 2 wk 3 wk

M1, primary motor cortex; SO, contralateral supraorbital area; tDCS, transcranial direct current stimulation.

##### 3.3.2.2. Stimulation parameters

Different types of tDCS devices and the types of electrodes were used across the included studies (Table 1). Five of the included studies^1,2,12,30,44^ used sponge electrodes of the size of 35 cm^2^ (ie, 5 × 7 cm), 3^9,46,50^ used sponge electrodes of 35 cm^2^ but did not report the details of length and width, and 2^5,45^ used electrodes of 16 cm^2^ (only one of them^45^ reported the actual length and width [ie, 4 × 4 cm]). The target current intensity of tDCS used in 8 studies^1,2,5,12,30,44,46,50^ was set at 2 mA, while other 2 studies^9,45^ were set at 1 mA. In 9 studies, the length of stimulation session was 20 minutes,^1,2,5,9,30,44–46,50^ and in one study,^12^ each session was 30 minutes.

##### 3.3.2.3. Stimulation protocol

All the included studies implemented not single, but multiple sessions of tDCS (session number = 5–16). The number of stimulations per week ranged from 2 to 5 times, and the whole stimulation duration ranged from 1 to 8 weeks. Eight studies^1,2,5,12,30,44,45,50^ implemented 5 times a week of stimulation for 1 to 3 weeks, one study^46^ used 3 times a week of stimulation for 2 weeks, and the other study^9^ used 2 times a week of stimulation for 8 weeks.

Both tDCS-only (n = 4)^1,5,30,50^ and tDCS in combination with other intervention (n = 6)^2,9,12,44–46^ were used. Specifically, 2 studies^2,44^ combined tDCS with mindfulness-based meditation (MBM); 2 studies^9,46^ combined tDCS with strengthening exercise; one study^12^ combined tDCS with sham muscle electrical stimulation (sham-EIMS); and one study^45^ combined tDCS with a battery of physiotherapy (including TENS, ultrasound, infrared heat, patellofemoral and tibiofemoral mobilization, and exercise therapy).

For the design of the control, inactive sham stimulation was used in 7 studies, that is, the electrodes in sham were placed in the same positions as tDCS, but the current was delivered for only the first 30 seconds of the stimulation.^1,2,5,9,12,44,50^ Uniquely, one study used TENS as the control.^46^ The participants from 2 studies randomly received one of the 4 protocols (Table 1).^30,45^

In 3 studies,^2,30,44^ the intervention was completed within the home of participants as remotely administrated by using a videoconference platform. In other 7 studies,^1,5,9,12,45,46,50^ the intervention was completed within the laboratory/clinical environment.

##### 3.3.2.4. Outcomes

The pain severity was assessed by the scores of NRS (n = 4)^1,2,30,44^ or VAS (n = 6).^5,9,12,45,46,50^ The score of NRS ranged from 0 to 100 in 4 studies.^1,2,30,44^ The score of VAS ranged from 0 to 100 in 2 studies,^9,46^ and from 0 to 10 in 3 studies.^5,12,45,50^

All studies assessed the pain severity before and after the intervention. Four of them also measured the pain severity in the middle of the intervention. Specifically, one study^1^ measured the pain severity before and after each of 5 intervention sessions; 2 studies^2,12^ measured after each session; and one study^50^ assessed after the 5th and 10th intervention session. The follow-up effects of tDCS were examined in 6 studies^1,5,30,45,46,50^ with a follow-up period between 1 week and 3 months.

##### 3.3.2.5. The effects of transcranial direct current stimulation on clinical pain intensity

In 7 of 10 studies,^1,2,12,30,44,45,50^ it was observed that tDCS induced significant reduction in KOA-related pain as compared to sham (Table 1). Inconsistently, in 2 studies,^5,9^ it was observed that both tDCS and sham stimulation induced significant reduction in pain intensity; in another study,^46^ no significant effects of tDCS on KOA-related pain compared to sham was observed.

##### 3.3.2.6. Blinding efficacy

Two studies^9,50^ reported successful blinding via the responses to designed blinding efficacy questionnaires; 6 studies^1,2,30,44–46^ did not report blinding efficacy; and 2 studies^5,12^ only provided vague description of blinding (eg, “seemed to have blinded patients effectively).”

##### 3.3.2.7. Side effects and attrition

Five studies^1,2,30,46,50^ reported no adverse events induced by tDCS. One study^5^ reported occasional itching in 3 patients from tDCS group and one from sham group, and skin irritation in one participant undergoing tDCS. One study^9^ reported 2 cases of side effects potentially due to tDCS, and one of them withdrew from the study. The other 3 studies^12,44,45^ did not report relevant information.

##### 3.3.2.8. Meta-analysis

One study^5^ did not report the mean and SD of the outcomes, and its authors did not respond to email query. Therefore, 9 of the 10 studies, expect for this one,^5^ were included in the meta-analysis.

##### 3.3.2.9. Effect of transcranial direct current stimulation on the intensity of knee osteoarthritis–related pain

**Figure 2.**
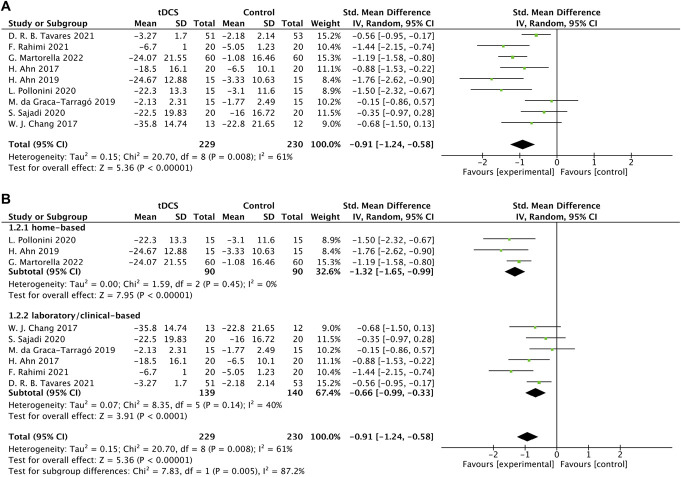
Forest plots.

Generally, tDCS induced significant improvements in the intensity of KOA-related pain as assessed using the scores of NRS or VAS as compared to control (SMD = −0.91, 95% CI [−1.24, −0.58], *P* < 0.001, I^2^ = 61%) (Fig. 2A).

##### 3.3.2.10. Subgroup analyses

We performed a series of subgroup analyses to explore the characteristics in study protocol that may influence the efficacy of tDCS, including stimulation environment (ie, home-based intervention [n = 3] and laboratory/clinical-based intervention [n = 6]) (Fig. 2B), stimulation type (ie, tDCS-only [n = 3] and tDCS in combination with other interventions [n = 6]), stimulation frequency (ie, <5 times/week [n = 2] and = 5 times/week [n = 7]) and number (ie, <10 sessions [n = 3] and ≥10 sessions [n = 6]), intensity = 2 mA (n = 7) and = 1 mA (n = 2), the measure of pain severity (ie, NRS [n = 4] and VAS [n = 5]), and participant age (ie, mean ages ≥60 years [n = 5] and <60 years [n = 4]). We also compared the immediate (ie, measured immediately after the last session of intervention [n = 9]) and follow-up (n = 5) effects of tDCS.

The subgroup analyses (Table 5) showed that compared to sham, (1) the home-based tDCS, the intervention combined tDCS with others, and that with the frequency of 5 times per week and/or with a total number of at least 10 sessions can induce significantly large effect size (home-based tDCS: SMD = −1.32, 95% CI [−1.65, −0.99], *P* < 0.001, I^2^ = 0%; combined tDCS: SMD = −0.95, 95% CI [−1.49, −0.41], *P* < 0.001, *I*^2^ = 68%; 5 times per week: SMD = −1.02, 95% CI [−1.41, −0.64], *P* < 0.001, *I*^2^ = 65%; at least 10 sessions: SMD = −1.12, 95% CI [−1.51, −0.74], *P* < 0.001, *I*^2^ = 59%); (2) the laboratory/clinical-based tDCS, and the tDCS-only intervention was moderate effect size (laboratory/clinical-based: SMD = −0.66, 95% CI [−0.99, −0.33], *P* < 0.001, I^2^ = 40%; tDCS-only: SMD = −0.88, 95% CI [−1.30, −0.45], *P* < 0.001, *I*^2^ = 60%); and (3) that with current intensity of both 1 and 2 mA induced significantly large effect size (1 mA: SMD = −1.09, 95% CI [−1.83, −0.35], *P* = 0.004, *I*^2^ = 48%; 2 mA: SMD = −0.87, 95% CI [−1.26, −0.48], *P* < 0.001, *I*^2^ = 67%). Meanwhile, such large effect size of tDCS was observed only immediately after the stimulation in both older participants and those under 60 years (older: SMD = −0.81, 95% CI [−1.25, −0.38], *P* < 0.001, *I*^2^ = 65%; younger: SMD = −1.06, 95% CI [−1.67, −0.46], *P* < 0.001, *I*^2^ = 66%). A large effect size and a moderate effect size were observed when using NRS and VAS as the outcome measure, respectively (VAS: SMD = −0.62, 95% CI [−1.01, −0.23], *P* = 0.002, *I*^2^ = 48%; NRS: SMD = −1.23, 95% CI [−1.53, −0.94], *P* < 0.001, *I*^2^ = 1%). No significant effect size was observed in any other protocol characteristics (Table 5).

**Table 5:** Results of subgroup analyses.

Category	Group factors	No. of studies	SMD (95% CI)	*P*	Test of heterogeneity		
					χ^2^	*P*	I^2^ (%)
Environment	Home-based or laboratory/clinical-based intervention						
	Laboratory/clinical-based	6	SMD −0.66 (−0.99, −0.33)	<0.001	8.35	0.14	40%
	Home-based	3	SMD −1.32 (−1.65, −0.99)	<0.001	1.59	0.45	0%
Comparison	tDCS-only intervention or tDCS combined with other interventions						
	tDCS-only	3	SMD −0.88 (−1.30, −0.45)	<0.001	5.04	0.08	60%
	tDCS combined with other interventions	6	SMD −0.95 (−1.49, −0.41)	<0.001	15.66	0.008	68%
Frequency	Frequency (weekly)						
	<5 times/week	2	SMD −0.47 (−0.97, 0.02)	0.06	0.42	0.52	0.00%
	5 times/week	7	SMD −1.02 (−1.41, −0.64)	<0.001	17.15	0.009	65%
	Number of sessions						
	<10 times	3	SMD −0.47 (−0.89, 0.05)	0.03	2.42	0.3	17%
	≥10 times	6	SMD −1.12 (−1.51, −0.74)	<0.001	12.22	0.03	59%
tDCS protocols	Current intensity						
	2 mA	7	SMD −0.87 (−1.26, −0.48)	<0.001	17.93	0.006	67%
	1 mA	2	SMD −1.09 (−1.83, −0.35)	0.004	1.92	0.17	48%
Characteristics of participant	Mean age						
	≥60	5	SMD −0.81 (−1.25, −0.38)	<0.001	11.3	0.02	65%
	<60	4	SMD −1.06 (−1.67, −0.46)	<0.001	8.84	0.03	66%
Follow-up	Immediate or follow-up						
	Immediate	9	SMD −0.91 (−1.24, −0.58)	<0.001	20.7	0.008	61%
	Follow-up	5	SMD −0.62 (−1.30, 0.07)	0.08	35.73	0	89%
VAS/NAS							
	VAS	5	SMD −0.62 (−1.01, −0.23)	0.002	7.77	0.1	48%
	NRS	4	SMD −1.23 (−1.53, −0.94)	<0.001	3.03	0.39	1%

NRS, Numeric Rating Scale; SMD, standardized mean difference; tDCS, transcranial direct current stimulation; VAS, Visual Analogue Scale.

##### 3.3.2.11. Publication bias

**Figure 3.**
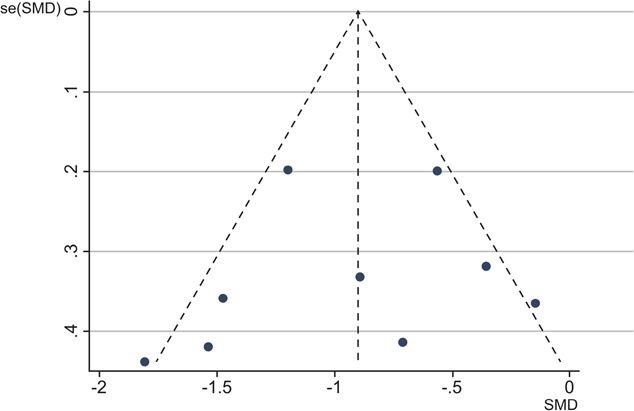
Funnel plot.

The funnel plots (Fig. 3) and Egger tests (*t* = −0.52, *P* = 0.616) indicated that there was no evidence of symmetry, suggesting no substantial publication bias on the results.

## 4. Discussion

To our knowledge, this work for the first time explicitly characterize the contributions of different factors to the effects of tDCS on the severity in KOA-related pain. Specifically, the results show that compared to control, both home- and laboratory-based tDCS with at least 5 sessions per week can induce significant benefits for KOA-related pain. These observations suggest that tDCS may help alleviate the pain symptom in people suffering from KOA, which needs to be confirmed by more studies.

We observed that tDCS targeting M1 can significantly alleviate KOA-related pain. This is in line with the findings from previous studies^26^ and review work^57^ that using tDCS targeting M1 can effectively alleviate the pain severity. Studies have proposed several conceptual models that may underneath such benefits of tDCS for pain alleviation. In the network effect theory, for example, tDCS can upregulate and downregulate functional connections between different brain regions,^11,28^ including those closely pertaining to pain (eg, cortical cognitive and sensorimotor regions).^23,43,55^ Meanwhile, in the synapse remodeling theory, tDCS can regulate the synaptic microenvironment and modulate neuronal function at the synaptic level, which interacts with several neurotransmitters, including dopamine, and acetylcholine and g-aminobutyric acid (GABA),^34–36,38^ and influences neuronal membrane channels, such as sodium and calcium ions.^26^ The tDCS-induced modulation in these elements can thus lead to the changes in excitability of nociceptive neurons that alters with the perception of pain.^7,8,16,21,22,31,49^ In addition, studies also suggest that tDCS can alleviate pain by modulating the thalamic inhibitory network and interfering with the cortex-cortical and cortex-subcortical synaptic connections related to the formation of pain.^6,27^ These theories provide conceptual pathways through which tDCS may help alleviate the pain. Only one study^11^ examined resting state data of patients with fibromyalgia using functional magnetic resonance imaging, more neuroimaging evidence is needed to understand how tDCS-induced changes in related regions/networks are linked to the tDCS-induced changes in pain. It is worthwhile to be explored in future studies using advanced neuromodeling techniques to navigate the current flow of tDCS to the specific targeting regions and neuroimaging technique to explicitly characterize the change within those targets as induced by tDCS.

Large variance in study designs was observed across the included studies. Our subgroup analyses showed that tDCS with 5 sessions per week can induce large and significant effect size. These results are consistent with previous studies^26^ showing that the effect of tDCS can be cumulated using greater number of stimulation sessions. Moreover, multiple factors may influence the appropriate number of stimulation sessions, such as the targeting regions, the severity of the condition, and the parameters of the current (eg, intensity).^25,26,37^ It is thus highly demanded to characterize the dose–response relationship of tDCS to the alleviation of KOA-related pain, obtaining the appropriate dose of tDCS that can induce maximum benefits.

Although studies showed that the tDCS-induced improvements can sustain to a certain time (eg, 2 weeks^1^), we here showed that the effect size of such follow-up improvement is not significant. This may be due to the high heterogeneity between studies, which thus should be taken with caution. Uniquely, we observed that tDCS can benefit both older and younger than 60 years participants, suggesting that age may not significantly alter with the benefits of tDCS for KOA-related pain. It should also be noted that although the choice of tDCS montage is critical to its effects, the number of publications using different montages of tDCS is very limited (eg, only one study used electrode size with 16 cm^2^, and all the studies used tDCS with 1 or 2 mA current intensity targeting M1). We were thus not able to perform subgroup analysis to explicitly compare the effects between different tDCS montages (eg, electrode type, size, polarity, placement, and current intensity) and different controls (eg, active sham vs inactive sham). More studies are thus needed to examine and compare the influences of different montages/parameters of tDCS on its possible benefits for KOA-related pain, especially the optimal intervention frequency and stimulation target of tDCS that can maximize its longer-term effects on KOA-related pain.

One important observation here is that the home-based tDCS intervention can induce comparable or even larger effects than the laboratory-based intervention. It is still in debt if tDCS can be effectively used in the home environment. Fregni et al. showed that, for example, home-based tDCS cannot induce benefits for people suffering from unilateral drug-resistant central/peripheral neuropathic pain who previously received repetitive transcranial magnetic stimulation (rTMS)^17^; while in the review paper of Pacheco-Barrios and colleagues, it suggested that home-based tDCS may still be helpful.^40^ The feasibility and efficacy of home-based tDCS intervention is critical to individuals who are inaccessible to clinics and laboratory (eg, living in the rural area). Our observation here suggests that the home-based tDCS would be a feasible intervention to at least benefit people with KOA-related pain. Future studies using advanced techniques (eg, cloud-based server to transform predesigned stimulation montage and the wireless current delivery techniques) are needed to establish feasible and standardized protocol for the home-based tDCS, which will ultimately expand the benefits of this technique to more populations in need.

The results of this work should still be taken with caution. First, the number of included studies is relatively small, and most of the included studies consisted of small sample size of participants, lowering the evidence level. Second, 8 of the 10 included studies used a double-blinded design, yet only 2 of them reported blinding efficacy and claimed that the blinding was successful. It is thus strongly encouraged for future studies to assess this important aspect, which may help determine the efficacy of the tDCS intervention. Third, several critical information of participants is missing, such as the use of medication and the severity of KOA, thus limiting the possibility to explicitly examine the influences of these characteristics on the observed effects of tDCS and increasing the risk of bias in the observations. Fourth, all studies used the conventional tDCS stimulation with large sponge electrodes (with electrode size was 35 cm^2^ or 16 cm^2^), which may induce diffused electrical field that interferes with its effects. Studies using novel high-definition tDCS with much greater focality of stimulation are needed.^32^ This work primarily focused on pain severity. More studies are needed to explore the effects of tDCS on the functional mobility, quality of life, and other aspects that are closely associated with pain. In addition, it was observed in 2 studies that the pain was alleviated in sham (ie, control) group,^5,9^ indicating potential placebo effects. This may be potentially due to the stimulation protocol.^53^ Studies have shown that the traditional inactive sham may produce significant analgesic effects^11^ (eg, the release of endogenous opioids).^14^ This thus suggests that more appropriate protocol of sham stimulation is needed, such as the acti-sham that is developed with the help of advanced neuromodeling techniques^33^ to improve the blinding efficacy and avoid the potential biophysiological effects of the traditional inactive sham stimulation on cortical excitability.

Taken together, this systematic review and meta-analysis demonstrate the potential of using tDCS to alleviate KOA-related pain. More studies with more rigorous designs and using advanced technologies of tDCS are needed to explore the appropriate protocol characteristics (eg, stimulation target, intervention frequency, etc.) that can maximize such benefits of tDCS for KOA-related pain.

## Disclosures

The authors have no conflict of interest to declare.

## Supplemental digital content

Supplemental digital content associated with this article can be found online at http://links.lww.com/PR9/A266.

## Supplementary Material

SUPPLEMENTARY MATERIAL
