# Supplementary material for: Transcranial direct current stimulation alleviates the pain severity in people suffering from knee osteoarthritis: a systematic review and meta-analysis
Source: Pain Rep. 2024 Dec 9;10(1):e1215. doi: 10.1097/PR9.0000000000001215 (PMC11630987; doi:10.1097/PR9.0000000000001215)
Supplement: SUPPLEMENTARY MATERIAL [file painreports-10-e1215-s001.pdf]

Supplementary Materials

The quality of the evidence (GRADE)

No of studies	Design	Risk of bias	Inconsistency	Indirectness	Imprecision	Other considerations	Absolute Effect (95% CI)	Quality	Importance
**Pain severity**									
9	randomized trials	serious[1]	serious[2]	no serious indirectness	no serious imprecision	none	SMD 0.91 higher (0.58 to 1.24 higher)	⊕⊕OO LOW	CRITICAL

[1] Four studies did not report allocation concealment and two studies did not report blinding (assessors blinded).
[2] moderate heterogeneity (I^2 = 61%).
